# Healthcare resource use in schizophrenia, EuroSC findings

**DOI:** 10.1080/20016689.2017.1372027

**Published:** 2017-09-05

**Authors:** A. Millier, M. Horváth, F. Ma, K. Kóczián, A. Götze, M. Toumi

**Affiliations:** ^a^ Health Economics and Outcomes Research, Creativ-Ceutical, Paris, France; ^b^ Market Access, Medical & Marketing, Gedeon Richter Plc, Budapest, Hungary; ^c^ Health Economics and Outcomes Research, Creativ-Ceutical, Beijing, China; ^d^ Public Health Department, Aix-Marseille University, France

**Keywords:** Schizophrenia, resource use, hospitalisation, outpatient visits, negative symptoms, EuroSC

## Abstract

**Background**: It is unclear if the burden associated with schizophrenia is affected by the type and severity of patient’s symptoms.

**Objective**: This study aims to quantify healthcare resource use associated with different profiles of schizophrenia symptoms.

**Study design**: Post-hoc analysis of data from a naturalistic follow-up study.

**Setting**: Secondary psychiatric services in France, Germany and the UK.

**Patients**: EuroSC cohort:, representative sample of 1,208 schizophrenia patients

**Main outcome measure**: We classified patients into eight health states, according to the Lenert classification (HS1–HS8), and estimated 6-month healthcare resource use (outpatient and day clinic visits, and hospitalisations) across the health states.

**Results**: Approximately half of the patients were classed as having mild symptoms (HS1), with around 20% experiencing moderate, predominantly negative symptoms (HS2). The remaining health states were represented by <10% of patients each. Very few patients experienced extremely severe symptoms (HS8). No health state was associated with excess utilisation across all resource types. In terms of outpatient visits, patients were estimated to see a psychiatrist most often (3.01–4.15 visits over 6 months). Hospital admission was needed in 11%(HS1) – 35%(HS8) of patients and inpatient stays were generally prolonged for all health states (39–57 days). The average number of inpatient days was highest for patients in HS8 (18.17 days), followed by patients with severe negative symptoms (HS4; 13.37 days). In other health states characterised by severe symptoms (HS5–HS7), the average number of inpatient days was approximately half of those seen for HS4 (6.09–7.66).

**Conclusion**: While none of the symptom profiles was associated with excess resource usage, hospitalization days were highest for HS with severe, predominantly negative or extremely severe symptoms. Patients with predominantly negative, moderate or severe symptoms appeared to have a high number of psychologist visits – an interesting finding that may reflect a specific therapeutic approach to the treatment of these patients.

## Background

Schizophrenia is a severe mental disorder, characterised by disintegration of thought processes and impaired emotional responsiveness [1]. The disease most commonly manifests itself through auditory hallucinations, paranoid or bizarre delusions, and disorganised speech and thinking; it is also generally accompanied by significant social and/or occupational dysfunction [2]. Schizophrenia is estimated to affect over 21 million people worldwide [3] and five million in the European Union (EU) [4], with an estimated 12-month prevalence of 1.2% amongst adults in the EU [4].

Diagnosis of schizophrenia is based on observed behaviour and patient-reported experiences [5]. Symptoms typically first occur in young adulthood [6] and are usually classed as positive (i.e. those that occur in patients with schizophrenia but not in unaffected individuals) and negative (i.e. those that are lacking in people affected by the disease, but are commonly found in others) [7]. Examples of positive symptoms include hallucinations and delusions, while social withdrawal, lack of motivation and diminished emotional reactivity are considered negative symptoms [8]. Schizophrenia is also often associated with cognitive deficits that may affect functions such as working memory and attention [8].

Clinical, as well as informal care is important in schizophrenia and the disease is associated with both extensive healthcare resource use and considerable caregiver involvement. In the EU, the average number of inpatient admissions due to schizophrenia, schizotypal and delusional disorders is estimated at 1.37 per every 1,000 people, with an average length of stay of 38.5 days [9] – over five times longer than the average length of stay following an acute myocardial infarction (7.3 days) [10]. Patients in nearly all EU countries are also offered day clinic and outpatient visits, in line with a growing trend to provide community-based mental health services [9].

In addition to the substantial healthcare resource use, patients with schizophrenia also require intensive informal care impacting several domains of the carers’ lives [11]. The intensity of care provided relates closely to perceived burden, with those providing more hours of care per week and being the only caregivers reporting higher levels of burden [11]. However, relevant reviews note that caregiver burden depends not only on the carer’s circumstances, but is also particularly associated with the patient experiencing positive symptoms (although negative symptoms also add to the burden) [12], and thought to increase with increasing symptom severity [13]. It can be reasonably expected that healthcare resource use is also linked to symptom type and severity. This study aims to quantify healthcare resource use associated with different profiles of schizophrenia symptoms. We used the European Schizophrenia Cohort (EuroSC) – a naturalistic 2-year follow-up of a cohort of 1,208 European schizophrenia patients [14]. Indeed, the EuroSC cohort has been extensively used in past research, which focused on investigating treatment efficacy [15], patients’ quality of life [16–18], employment [19], social contact [20,21] and subjective feelings of security and safety [22], as well as assessing caregiver burden [23] and quantifying direct health care costs associated with managing the disease [24,25].

We used the Lenert classification [26,27] to determine the type and severity of schizophrenia symptoms experienced by EuroSC patients. The Lenert classification was developed to quantify how different symptom profiles impact the quality of life of those affected by schizophrenia [26]. Briefly, this was achieved by assigning different types of schizophrenia symptoms (positive, negative and cognitive) to eight health states (HSs) encompassing the full spectrum of overall symptom severity, from mild, through moderate and severe, to extremely severe [26]. Despite being developed in the US [26], the Lenert classification may be generalisable to the European population [28]. Indeed, the Lenert classification of HS and the corresponding utility weights have been used in a number of pharmacoeconomic analyses in European and non-European countries [29–34]. The present study provides estimates of resource use associated with schizophrenia management for each of the eight Lenert HS. This quantification of healthcare requirements of patients with different symptom characteristics is likely to assist future pharmacoeconomic studies focused on schizophrenia.

## Methods

### Data source

The data used in this publication were obtained from the EuroSC cohort – a naturalistic study of 1,208 schizophrenia patients aged 18–64 from France (N = 288), Germany (N = 618) and the UK (N = 302) [14]. The EuroSC study aimed to collect information on schizophrenia treatment and care, and investigate the relationship between disease management and clinical outcomes [14], quality of life [17], and other aspects of living with schizophrenia. Patients were followed for a total of 2-years, and, during that time, data were collected at 6-monthly intervals when patients attended their five interview sessions – the first one at baseline and the following 4 every 6 months thereafter [14]. Three study centres were located in France, two in the UK and four in Germany [14], and the study included a representative sample of patients treated in secondary psychiatric services in each country. Those who had been hospitalised for 12 months prior to enrolment, were roofless, or expected to move away from the area during the study period were excluded [14]. All participants provided written informed consent [14]. Study centres and patient sampling procedures were aligned with the organisation of mental health services in each country and random sampling was used where possible [14].

### Data collected

The EuroSC study involved numerous assessments at each visit [14]; however, the present study only considered patient characteristics (e.g. age and gender) and their clinical symptoms, healthcare resource use, and caregiver burden.

Information on healthcare resource use was collected from patient’s key workers using the standardised Client Service Receipt Interview (CSRI) [35] and the Malin System [36]. A range of data was collected [14]; those relevant to the current study included the type of service, and the frequency and duration of attendance.

Assessment of the patient’s clinical state was performed using the Positive and Negative Syndrome Scale (PANSS) [37,38], which includes 30 items, scored on a seven-point severity scale, that provide an overall score for positive symptoms (seven items) and negative symptoms (seven items), and a general measure of psychopathology (16 items) [14]. The questionnaire is interviewer-administered, requiring a 30–40 minute semi-formal psychiatric interview [38]. Responses are based on patient experiences over a pre-specified period preceding the interview, usually 1 week [38].

PANSS scores were used by Lenert *et al*. [26] to group patients with schizophrenia into eight health states, based on the type and severity of symptoms experienced. Disease symptoms were grouped into three factors: negative (PANSS items G7, G16, N1–4 and N6), positive (G9, P1, P3, P5 and P6) and cognitive (G5, G10–13, G15, P2 and N5) [26]. Each of the eight HSs corresponded to a range of scores for the three factors, resulting in HSs characterised by discrete symptom profiles (with State 1 described as mild symptoms and State 8 as extremely severe symptoms) [26].

### Statistical analysis

We computed descriptive statistics, including the mean number of visits or inpatient days and the corresponding standard deviation (SD), for the following healthcare resources used during the 6-month period preceding each of the five study visits: consultations with general practitioner (GP), psychologist, psychiatrist and other specialists, day-clinic visits and the length of inpatient stay.

Use of health services by patients suffering from schizophrenia over a 6-month period was estimated using a two-part statistical model based on two generalised linear mixed models (GLMMs). The model assumed that the amount of resource utilised results from a combination of two items: the decision/necessity to use the resource (or not), and the amount of resource used, which is conditional upon consuming any resource. The first GLMM used a logistic link function to model the odds of utilising a given type of resource (e.g. the probability of seeing a psychiatrist) based on patient characteristics (age, gender and Lenert HS). The second GLMM modelled the amount of resource consumed given that the patient used this type of resource (e.g. the number of psychiatrist visits per patient with at least one visit), as a function of patient characteristics. As resource use values usually have an asymmetric right-skewed distribution, several distributions were tested to identify one that fitted the observed data best, including Poisson, negative binomial, gamma and log-normal distributions. Based on minimisation of Akaike Information Criterion (AIC) [39,40], for all types of resources gamma distribution was selected as the most appropriate. Results from the two parts of the model were combined by multiplying the probability of using a given resource type (from Part I) and the amount used (from Part II) to obtain average resource consumption for a representative patient in each Lenert health state.

No missing data replacement was scheduled.

## Results

### Patient disposition

As seen in Table 1, which presents patient disposition, throughout the duration of the study about 50% of EuroSC patients were classed as fitting the description of Lenert HS1, corresponding to mild symptoms of schizophrenia. HS2, characterised by predominance of negative symptoms and moderate severity, described the condition of approximately 20% of patients. In contrast, only about 10% of patients experienced a mixture of moderate positive and negative symptoms (HS3). Amongst patients with severe symptoms (HS4–HS7), states where negative (HS4) or negative and cognitive (HS6) symptoms were dominant were more common than those with a predominance of positive and cognitive (HS5) or positive (HS7) symptoms.

**Table 1.** Patient disposition.

	Visit 1 (N = 1163) n (%)	Visit 2 (N = 981) n (%)	Visit 3 (N = 899) n (%)	Visit 4 (N = 816) n (%)	Visit 5 (N = 782) n (%)
HS 1 (mild symptoms)	588 (50.56%)	499 (50.87%)	456 (50.05%)	414 (50.74%)	418 (53.45%)
HS 2 (moderate, negative dominance)	228 (19.60%)	212 (21.61%)	195 (21.36%)	188 (23.04%)	174 (22.25%)
HS 3 (moderate, positive & negative)	104 (8.94%)	93 (9.48%)	102 (11.17%)	79 (9.68%)	73 (9.34%)
HS 4 (severe, negative dominance)	95 (8.17%)	87 (8.87%)	85 (9.31%)	65 (7.97%)	62 (7.93%)
HS 5 (severe, positive & cognitive)	41 (3.53%)	31 (3.16%)	24 (2.63%)	15 (1.84%)	11 (1.41%)
HS 6 (severe, negative & cognitive)	53 (4.56%)	18 (1.83%)	23 (2.52%)	24 (2.94%)	22 (2.81%)
HS 7 (severe, positive dominance)	40 (3.44%)	31 (3.16%)	21 (2.30%)	22 (2.70%)	17 (2.17%)
HS 8 (Extremely severe symptoms)	14 (1.20%)	10 (1.02%)	6 (0.66%)	9 (1.10%)	5 (0.64%)

HS = Health State

*Presents the number of patients in each of the eight Lenert health states at a given study visit. Note different total patient numbers at each study visit, reflecting loss to follow-up.*

### Resource use

**Figure 1.**
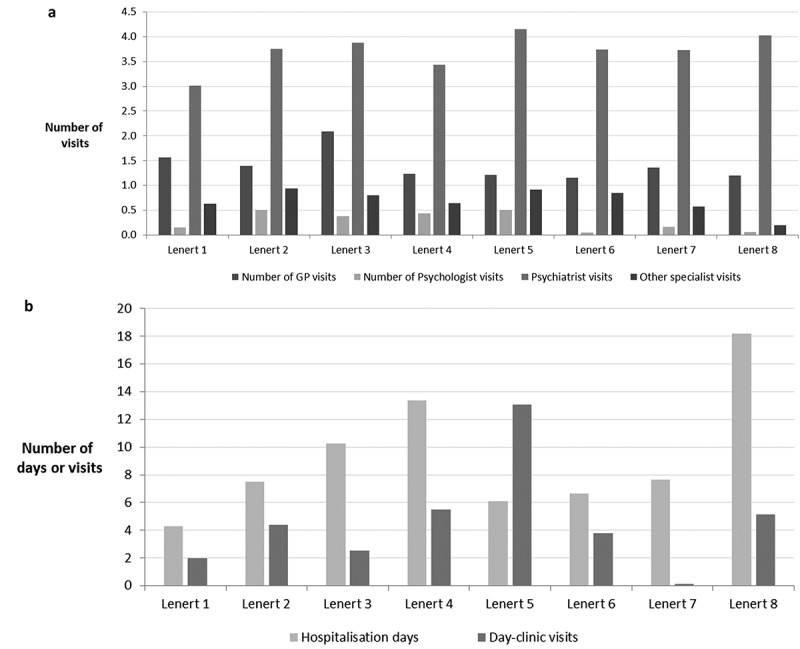
Estimated resource use over 6 months, by Lenert health state.

Resource use in the EuroSC cohort was estimated from the raw data (Supplementary Table 1) using the two-part statistical model described in the methods. Table 2 presents the estimated probability of using a given type of resource (Part I) and resource utilisation in those who used the service (Part II). The final result provides estimated 6-month resource use for a representative patient in each Lenert health state (Figure 1). Details of multivariate regression used to obtain these results are presented in Supplementary Table 2.

**Table 2.** Resource use over 6 months – multivariate results.

	GP			Psychologist visits			Psychiatrist visits			Other specialist visits			Day-clinic visits			Hospitalisation days		
	Part I	Part II	Final	Part I	Part II	Final	Part I	Part II	Final	Part I	Part II	Final	Part I	Part II	Final	Part I	Part II	Final
HS 1 (mild symptoms)	50.61%	3.09	**1.56**	2.91%	5.32	**0.15**	81.03%	3.71	**3.01**	24.15%	2.63	**0.63**	4.31%	46.10	**1.98**	10.50%	40.90	**4.29**
HS 2 (moderate, negative dominance)	42.54%	3.27	**1.39**	4.31%	11.69	**0.50**	81.99%	4.57	**3.75**	21.55%	4.34	**0.93**	7.51%	58.63	**4.40**	16.42%	45.57	**7.48**
HS 3 (moderate, positive & negative)	47.91%	4.35	**2.08**	3.72%	10.08	**0.37**	76.98%	5.03	**3.87**	20.59%	3.90	**0.80**	5.69%	44.48	**2.53**	21.18%	48.37	**10.25**
HS 4 (severe, negative dominance)	33.44%	3.70	**1.24**	3.60%	12.08	**0.44**	80.01%	4.29	**3.43**	15.10%	4.22	**0.64**	11.24%	48.95	**5.50**	23.29%	57.43	**13.37**
HS 5 (severe, positive & cognitive)	31.09%	3.89	**1.21**	3.30%	15.30	**0.51**	79.18%	5.24	**4.15**	17.72%	5.18	**0.92**	14.26%	91.68	**13.07**	15.12%	40.31	**6.09**
HS 6 (severe, negative & cognitive)	33.99%	3.39	**1.15**	0.79%	6.02	**0.05**	78.50%	4.76	**3.74**	9.67%	8.78	**0.85**	9.25%	41.08	**3.80**	17.14%	38.71	**6.63**
HS 7 (severe, positive dominance)	43.40%	3.13	**1.36**	1.63%	10.07	**0.16**	71.67%	5.21	**3.73**	14.56%	3.93	**0.57**	1.62%	7.91	**0.13**	15.03%	50.94	**7.66**
HS 8 (Extremely severe symptoms)	35.35%	3.40	**1.20**	2.16%	2.90	**0.06**	72.93%	5.52	**4.03**	14.20%	1.36	**0.19**	8.34%	61.34	**5.12**	34.75%	52.29	**18.17**

GP = General Practitioner, HS = Health State

*Considers mean age of 41.87 years and 61.81% of males, as per EuroSC results. Part I provides the probability of using the given resource type for a patient in each health state and Part II the extent of use amongst service users. Final result is the estimate of resource consumption for a representative patient in each health state.*

Unsurprisingly, patients were most likely to visit a psychiatrist (70–80% probability, depending on health state) compared with other types of physician visits analysed in this study (Table 2, Figure 1(a)). Those with mild symptoms (HS1) appeared to require fewest visits, which was evident both amongst those with at least one visit and amongst all patients (Table 2). Compared with psychiatrist visits, patients were substantially less likely to visit other specialists (probability of 10–20%) and the number of visits amongst service users varied widely, ranging from two to nine. On average, for all health states the number of other specialist visits per patient was low (<1). Patients in HS2, HS5 and HS6 appeared to require most visits to non-psychiatric specialists; however, the reasons for this are not entirely clear. In general, few patients appeared to visit a psychologist (0–15%), which resulted in an average of less than one visit per patient for all health states. However, those patients who did see a psychologist had between 3 and 15 appointments, depending on health state. Interestingly, in our study patients with predominantly negative, moderate (HS2) or severe (HS4) symptoms had some of the highest numbers of psychologist visits amongst all health states. With regard to the use of primary care resources, 30–40% of patients were estimated to visit their GP, with an average of 1–2 visits per patient (Table 2). Patients with moderate, positive and negative symptoms (HS3) required most GP visits amongst the eight health states, both when patients with at least one visits and all patients were considered.

Depending on health state, between 11% and 35% of patients were estimated to require hospital admission (Table 2 and Figure 1(b)). Patients with extremely severe symptoms (HS8) were at highest risk of hospitalisation (35%), while those with mild symptoms (HS1) were least likely to be admitted (10.5%). However, amongst hospitalised patients, inpatient stay was generally prolonged regardless of health state (39–57 days). Thus, reflecting the differences in admission probability, the average number of inpatient days for HS8 (18.17 days) was highest, while that for HS1 was lowest (4.29 days). Importantly, the second highest (after HS8) average number of inpatient days (13.37) was noted for patients with severe negative symptoms (HS4), reflecting a high admission risk (23.29%) combined with a long inpatient stay upon admission (57 days). The average inpatient stay for HS4 was approximately twice as long as for the other health states characterised by severe symptoms (HS5–HS7: 6.09–7.66), highlighting an increased risk of prolonged inpatient stay associated with severe negative symptoms.

Day clinic use varied widely between health states, both in terms of the patients’ probability of attending (2–14%) and the number of visits amongst those who did attend (7.91–91.68). Patients with severe, positive and cognitive symptoms (HS5) were estimated to require, on average, 13.07 admissions–over twice as much as those with severe, predominantly negative symptoms (HS4, 5.50 visits) or even those with extremely severe symptoms (HS8, 5.12 visits).

## Discussion

We used statistical modelling to estimate resource use associated with different profiles of symptoms that patients with schizophrenia may experience. We studied the EuroSC cohort, a representative sample of schizophrenia patients from three European countries, which has been extensively used in prior research on this debilitating disease [15–25] Within this cohort, up to a third of patients were in HS2 or HS4 and experienced predominantly negative symptoms that were classed as moderate or severe, in line with earlier studies suggesting that negative symptoms of schizophrenia are indeed very common. In previous reports, 41% of patients experienced two or more negative symptoms [41], while as many as 58% had at least one negative symptom [42]. The impact of negative symptoms on overall functional impairment is substantial and no specific treatment can be considered particularly effective against these symptoms, according to the evidence base summarised in a recent review [43]. Negative symptoms of schizophrenia may pose a particular burden to patients, due to their links to lower quality of life and reduced social contact – a recent study, which also used the EuroSC cohort, showed that social isolation in patients with schizophrenia is associated with poor psychosocial functioning and low quality of life (QoL) [21]. Importantly, low level of social activity also aggravates negative symptoms, which further reduce social interactions, forming a vicious circle reinforcing the disease [21].

One of the substantial advances of our study over earlier research is that it provides insights into resource use in patients with different profiles of schizophrenia symptoms. An earlier study by Heider *et al*. investigating resource use in the whole EuroSC cohort [24] reported results that were broadly similar to ours and to those obtained by Sarlon *et al*. in the subset of 288 French patients [25]. However, the figures estimated by Heider *et al*. [24] and Sarlon *et al*. [25] did not take into account the variability between different symptom profiles that was clear from our study, especially with regard to hospitalisation days, day-clinic visits and psychologist appointments. In our study, the vast majority (72–82%) of patients visited a psychiatrist multiple times over the 6-month period. This was similar to the results obtained by Sarlon *et al*. who showed that 80% of all patients visited a psychiatrist and the mean number of visits over 6 months amongst these patients was 6.05 [25], suggesting that the majority could be followed-up by their psychiatrist on a monthly basis. With regard to inpatient admissions, the length of hospital stay in our study was 39–57 days, depending on health state, with the longest duration of hospitalisation observed in those with a predominance of severe negative symptoms. This is somewhat higher than the average of 38.5 days estimated for the EU in a report prepared for the Executive Agency for Health and Consumers [9], possibly due to the fact that about a quarter of patients in our study were treated in the UK, where inpatient admissions are on average longer than in other European countries [9] – a difference which was also observed in the EuroSC cohort [24]. This finding is important to be highlighted as despite the patients with negative symptoms are not demanding nor disturbing the organisation, they end up being very expensive to medially manage.

In our study, no particular health state was associated with excess utilisation across all resource types. In terms of hospitalisations, which are likely to be the most expensive of the healthcare resources investigated, the average needs in patients with severe negative symptoms (HS4) were lower only than in those with extremely severe symptoms (HS8). These results suggest that effective targeting of negative symptoms is likely not only to improve quality of life and social interaction in patients with schizophrenia, as described above, but it may also substantially lower the burden schizophrenia poses on healthcare systems, especially given that negative symptoms are rather common [41,42].

Regarding the use of other resource types, patients in HS2, HS5 and HS6 appeared to require most visits to non-psychiatric specialists. These three health states are characterised by distinct symptom profiles (HS2 = moderate, predominantly negative symptoms; HS5 = severe with positive and cognitive symptoms; and HS6 = severe with negative and cognitive symptoms) and there appears to be no clear common factor that could prompt the patients to see other specialists. However, as our study did not account for comorbidities, it is unclear whether those visits were directly associated with the symptom profile characteristic of these health states, or whether patients in these states had higher prevalence of comorbidities (e.g. diabetes) requiring more frequent specialist visits.

Psychologist visits were the least frequently utilised resource type for nearly all health states. However, patients who attended these appointments generally did so several times over 6 months, possibly reflecting participation in structured therapy spanning multiple sessions. Interestingly, patients with predominantly negative, moderate (HS2) or severe (HS4) symptoms had, on average, some of the highest numbers of psychologist visits amongst all health states, potentially reflecting attempts to ameliorate negative symptoms using psychotherapeutic interventions, which have previously been reported to moderately improve negative symptoms [44]. It would be interesting to further investigate this apparent relationship between the predominance of negative symptoms and increased utilisation of psychological help.

Some of the limitations of our study pertain to the use of the EuroSC cohort. Although the sampling procedure for this cohort aimed to include a representative patient group, the EuroSC cohort included mostly patients with long-term episodic or continuous paranoid schizophrenia; few patients had only a single disease episode in full r partial remission [14]. However, patients with very severe forms of the disease – who may use healthcare resources quite intensively – were likely not included in the study, as it excluded those who had been hospitalised for 12 month prior to enrolment. Furthermore, data for the EuroSC cohort was collected between 1998 and 2002, so that any changes in mental health care brought about in the last 15 years by European and national initiatives [45] will not be captured in this study. However, the large sample size, long follow-up and comprehensive range of data available for this cohort partially overcome these limitations, making it a valuable population for real-world studies on schizophrenia.

Another limitation of our study is linked to the way resource use was defined and quantified. Notably, the study did not distinguish between hospitalisations for schizophrenia and those due to other reasons. Between-country variations in healthcare provision for patients with schizophrenia have been shown to exist in the EuroSC cohort [24], but these were not captured in our study. Furthermore, as mentioned above, comorbidities were not taken into account in this analysis. Patients with mental disorders are at greater risk of early mortality and physical illness, and have higher disability levels [9]. There is good evidence for increased risk of cardiovascular diseases (including stroke and myocardial infarction), obesity, metabolic syndrome, diabetes, hyperlipidaemia, obstetric complications, HIV, and a number of other health issues in those affected by a severe mental disorder [46]. Healthcare resource use related to managing these comorbidities and the associated costs are likely to pose a considerable burden on health services, but, in our study, these are not distinguishable from resource use due to schizophrenia itself.

Beyond the concrete findings of this study, it may re-open the collective thinking on more effective and efficient method on reallocation of public funding, by offering a more specific health-state based financing, including some major clinical differentiators for a better recognition of disease specificity and management [47].

A renewed resource allocation method for mental health may introduce some new dimensions, such as diagnostic and symptoms/severity, to ensure coordination of all health care practitioners around the patient needs. Incentive and/or quality metrics management would be especially relevant for patients with negative symptoms, who are not disturbing the psychiatric organisation and are not demanding like patients with positive symptoms exacerbations.

## Conclusion

This study modelled resource allocation in a longitudinal, representative sample of 1,208 European patients with schizophrenia, classified into eight distinct health states based on their symptom profile. While none of the symptom profiles was associated with excess usage of all resources tested, patients with severe negative symptoms had substantial hospitalisation needs that were the second-highest across all health states (lower only than in patients with extremely severe symptoms), suggesting that targeting these symptoms effectively may lower the negative impact of schizophrenia on the healthcare system. Further, patients with predominantly negative, moderate or severe symptoms appeared to have a high number of psychologist visits – an interesting finding that may reflect a specific therapeutic approach to the treatment of these patients.

## Supplementary Material

EuroSC_costs_supplement.docx
