# Supplementary material for: Healthcare resource use in schizophrenia, EuroSC findings
Source: J Mark Access Health Policy. 2017 Sep 5;5(1):1372027. doi: 10.1080/20016689.2017.1372027 (PMC5645906; doi:10.1080/20016689.2017.1372027)
Supplement: EuroSC_costs_supplement.docx [file ZJMA_A_1372027_SM7347.docx]

**Supplementary Table 1. Resource use over 6 months - descriptive results**

|  | **Visit 1**  **Mean (SD)** | **Visit 2**  **Mean (SD)** | **Visit 3**  **Mean (SD)** | **Visit 4**  **Mean (SD)** | **Visit 5**  **Mean (SD)** |
| --- | --- | --- | --- | --- | --- |
| **GP visits** |  |  |  |  |  |
| HS 1 | 1.41 (2.82) | 1.42 (2.87) | 1.62 (2.88) | 1.70 (2.67) | 1.80 (2.95) |
| HS 2 | 0.85 (1.82) | 1.43 (3.17) | 1.50 (2.72) | 1.70 (3.62) | 1.54 (2.46) |
| HS 3 | 1.36 (3.17) | 3.2 (10.19) | 2.15 (5.69) | 1.92 (3.02) | 1.85 (3.41) |
| HS 4 | 1.20 (3.98) | 1.18 (2.26) | 1.41 (2.35) | 1.25 (2.08) | 1.10 (1.68) |
| HS 5 | 0.34 (1.33) | 1.35 (3.25) | 2.17 (3.85) | 2.80 (7.35) | 1.18 (1.17) |
| HS 6 | 1.64 (3.76) | 0.94 (2.36) | 0.87 (1.63) | 0.67 (1.46) | 0.95 (1.73) |
| HS 7 | 0.45 (0.99) | 1.10 (1.58) | 2.29 (5.18) | 2.14 (2.80) | 2.00 (3.16) |
| HS 8 | 0.29 (0.83) | 1.8 (2.66) | 2.17 (3.92) | 0.22 (0.44) | 2.60 (2.79) |
| **Psychologist visits** |  |  |  |  |  |
| HS 1 | 0.14 (1.06) | 0.12 (0.95) | 0.20 (1.72) | 0.15 (1.32) | 0.24 (1.64) |
| HS 2 | 0.71 (4.99) | 0.47 (2.52) | 0.56 (3.21) | 0.46 (2.55) | 0.26 (1.54) |
| HS 3 | 0.19 (1.00) | 0.70 (3.31) | 0.57 (3.41) | 0.30 (2.7) | 0.01 (0.12) |
| HS 4 | 0.35 (2.55) | 0.18 (1.24) | 0.81 (3.91) | 0.02 (0.12) | 0.76 (3.64) |
| HS 5 | 0.34 (2.19) | 1.48 (7.05) | 0.00 (0.00) | 0.00 (0.00) | 0.00 (0.00) |
| HS 6 | 0.00 (0.00) | 0.00 (0.00) | 0.00 (0.00) | 0.00 (0.00) | 0.27 (1.28) |
| HS 7 | 0.00 (0.00) | 0.00 (0.00) | 0.29 (1.31) | 0.73 (3.41) | 0.00 (0.00) |
| HS 8 | 0.00 (0.00) | 0.00 (0.00) | 0.00 (0.00) | 0.33 (1.00) | 0.00 (0.00) |
| **Psychiatrist visits** |  |  |  |  |  |
| HS 1 | 3.38 (4.14) | 2.99 (3.84) | 3.06 (4.00) | 2.70 (3.08) | 2.92 (3.38) |
| HS 2 | 3.89 (4.96) | 4.20 (5.94) | 3.66 (5.22) | 3.39 (3.74) | 3.44 (5.23) |
| HS 3 | 4.24 (3.71) | 3.20 (5.40) | 3.63 (5.59) | 3.58 (5.83) | 4.62 (7.06) |
| HS 4 | 3.27 (3.20) | 3.99 (4.03) | 3.35 (2.83) | 3.37 (3.01) | 2.85 (2.9) |
| HS 5 | 4.34 (4.94) | 4.74 (3.83) | 3.21 (2.60) | 6.07 (5.04) | 1.27 (1.19) |
| HS 6 | 4.15 (4.16) | 4.39 (5.84) | 3.48 (4.20) | 3.21 (3.22) | 2.27 (2.00) |
| HS 7 | 3.68 (4.42) | 4.71 (8.49) | 5.05 (10.05) | 1.86 (2.25) | 2.00 (3.10) |
| HS 8 | 5.29 (7.64) | 4.00 (3.43) | 2.83 (5.08) | 2.78 (2.39) | 3.40 (2.51) |
| **Other specialist visits** |  |  |  |  |  |
| HS 1 | 0.39 (1.25) | 0.78 (3.87) | 0.84 (3.24) | 0.65 (1.61) | 0.80 (2.60) |
| HS 2 | 0.83 (4.16) | 0.85 (3.73) | 0.87 (4.18) | 1.38 (5.94) | 0.59 (1.33) |
| HS 3 | 0.36 (1.13) | 0.74 (3.37) | 0.82 (2.98) | 1.65 (5.21) | 0.89 (4.83) |
| HS 4 | 0.53 (2.61) | 0.55 (3.01) | 0.67 (2.72) | 0.42 (1.70) | 1.00 (5.14) |
| HS 5 | 1.17 (5.45) | 0.42 (1.39) | 1.92 (5.39) | 0.67 (1.68) | 0.18 (0.60) |
| HS 6 | 0.15 (0.86) | 0.61 (1.97) | 0.30 (1.26) | 1.58 (5.38) | 2.50 (11.50) |
| HS 7 | 0.10 (0.31) | 0.68 (2.88) | 2.38 (8.49) | 0.23 (0.69) | 0.00 (0.00) |
| HS 8 | 0.00 (0.00) | 0.10 (0.32) | 0.00 (0.00) | 0.89 (0.93) | 0.00 (0.00) |
| **Day-clinic visits** |  |  |  |  |  |
| HS 1 | 2.39 (13.02) | 3.59 (17.16) | 0.87 (6.78) | 1.41 (10.02) | 1.63 (10.15) |
| HS 2 | 4.74 (19.26) | 5.89 (23.44) | 5.42 (22.17) | 3.23 (15.26) | 3.09 (18.03) |
| HS 3 | 3.14 (18.7) | 4.27 (17.14) | 1.46 (10.42) | 3.19 (15.52) | 0.36 (3.04) |
| HS 4 | 5.27 (19.6) | 5.47 (22.15) | 7.04 (23.13) | 3.91 (14.91) | 5.50 (21.80) |
| HS 5 | 21.39 (44.79) | 10.94 (30.39) | 8.38 (26.72) | 0.00 (0.00) | 9.18 (30.45) |
| HS 6 | 4.11 (14.91) | 5.83 (19.26) | 1.13 (5.42) | 2.08 (7.06) | 7.09 (28.00) |
| HS 7 | 0.00 (0.00) | 0.00 (0.00) | 0.62 (2.84) | 0.00 (0.00) | 0.06 (0.24) |
| HS 8 | 9.43 (35.28) | 2.90 (9.17) | 0.00 (0.00) | 4.44 (13.33) | 8.00 (17.89) |
| **Hospitalisation days** |  |  |  |  |  |
| HS 1 | 5.35 (18.82) | 4.78 (17.22) | 4.38 (20.7) | 4.47 (19.63) | 3.05 (12.81) |
| HS 2 | 11.55 (28.89) | 9.57 (26.1) | 5.75 (20.51) | 5.40 (19.59) | 5.25 (21.57) |
| HS 3 | 12.66 (25.91) | 10.4 (34.04) | 10.61 (42.79) | 9.41 (20.96) | 7.60 (22.58) |
| HS 4 | 19.23 (40.76) | 17.98 (38.83) | 5.33 (15.43) | 11.92 (33.77) | 11.03 (34.90) |
| HS 5 | 5.80 (25.64) | 8.35 (24.75) | 8.75 (22.27) | 1.33 (5.16) | 0.64 (2.11) |
| HS 6 | 6.92 (19.83) | 1.78 (7.54) | 9.70 (40.63) | 8.00 (27.59) | 3.82 (11.31) |
| HS 7 | 6.98 (22.00) | 7.48 (25.45) | 1.95 (8.95) | 16.82 (46.75) | 4.82 (14.49) |
| HS 8 | 17.93 (40.91) | 33 (44.53) | 0.00 (0.00) | 28.11 (31.94) | 0.00 (0.00) |

GP = General Practitioner

**Supplementary Table 2. Resource use over 6 months - multivariate regressions**

|  | **GP** | | **Psychologist visits** | | **Psychiatrist visits** | | **Other specialist visits** | | **Day-clinic visits** | | **Hospitalisation days** | |
| --- | --- | --- | --- | --- | --- | --- | --- | --- | --- | --- | --- | --- |
| **PART I** | **Estimate** | **P-Value** | **Estimate** | **P-Value** | **Estimate** | **P-Value** | **Estimate** | **P-Value** | **Estimate** | **P-Value** | **Estimate** | **P-Value** |
| Intercept | -1.1361 | 0.0009 | -2.5609 | 0.0161 | 1.0095 | 0.0067 | -2.1099 | <.0001 | -0.9875 | 0.0901 | 0.3355 | 0.3532 |
| Age | 0.0158 | <.0001 | -0.0227 | 0.0037 | 0.0008 | 0.8187 | 0.0146 | <.0001 | -0.0297 | <.0001 | -0.0225 | <.0001 |
| Gender (male) | -0.2089 | 0.001 | -0.4909 | 0.0033 | -0.0840 | 0.2895 | -0.4858 | <.0001 | -0.2679 | 0.0367 | -0.0379 | 0.6729 |
| Gender (female) | - | - | - | - | - | - | - | - | - | - | - | - |
| HS 1 | 0.6281 | 0.051 | 0.3075 | 0.763 | 0.4611 | 0.1787 | 0.6542 | 0.1414 | -0.7046 | 0.1885 | -1.5130 | <.0001 |
| HS 2 | 0.3030 | 0.3531 | 0.7157 | 0.4846 | 0.5246 | 0.1323 | 0.5065 | 0.2594 | -0.1148 | 0.8314 | -0.9974 | 0.0023 |
| HS 3 | 0.5199 | 0.1193 | 0.5601 | 0.591 | 0.2166 | 0.544 | 0.4493 | 0.326 | -0.4112 | 0.466 | -0.6840 | 0.0419 |
| HS 4 | -0.0849 | 0.8015 | 0.5279 | 0.6146 | 0.3960 | 0.2735 | 0.0721 | 0.8768 | 0.3306 | 0.5481 | -0.5622 | 0.0958 |
| HS 5 | -0.1921 | 0.6093 | 0.4377 | 0.6994 | 0.3449 | 0.3964 | 0.2637 | 0.5994 | 0.6024 | 0.3064 | -1.0952 | 0.0071 |
| HS 6 | -0.0601 | 0.87 | -1.0125 | 0.4777 | 0.3044 | 0.4433 | -0.4356 | 0.4115 | 0.1132 | 0.8523 | -0.9459 | 0.0154 |
| HS 7 | 0.3381 | 0.3556 | -0.2863 | 0.8172 | -0.0629 | 0.8721 | 0.0293 | 0.9541 | -1.7113 | 0.0536 | -1.1021 | 0.0061 |
| HS 8 | - | - | - | - | - | - | - | - | - | - | - | - |
| **PART II** | **Estimate** | **P-Value** | **Estimate** | **P-Value** | **Estimate** | **P-Value** | **Estimate** | **P-Value** | **Estimate** | **P-Value** | **Estimate** | **P-Value** |
| Intercept | 1.0945 | <.0001 | 0.8772 | 0.3458 | 1.9213 | <.0001 | 0.1129 | 0.7808 | 3.7095 | <.0001 | 3.7390 | <.0001 |
| Age | 0.0035 | 0.0275 | 0.0052 | 0.4935 | -0.0040 | 0.0005 | 0.0070 | 0.0148 | 0.0063 | 0.2823 | 0.0039 | 0.3045 |
| Gender (male) | -0.0263 | 0.4642 | -0.0512 | 0.7555 | -0.0728 | 0.0056 | -0.1638 | 0.0079 | 0.2316 | 0.0462 | 0.0881 | 0.2598 |
| Gender (female) | - | - | - | - | - | - | - | - | - | - | - | - |
| HS 1 | -0.0965 | 0.6391 | 0.6077 | 0.4901 | -0.3968 | 0.0032 | 0.6616 | 0.0876 | -0.2857 | 0.5249 | -0.2455 | 0.3235 |
| HS 2 | -0.0402 | 0.8469 | 1.3955 | 0.1157 | -0.1890 | 0.1644 | 1.1625 | 0.003 | -0.0452 | 0.9207 | -0.1374 | 0.5861 |
| HS 3 | 0.2451 | 0.2465 | 1.2471 | 0.1676 | -0.0931 | 0.5041 | 1.0574 | 0.0078 | -0.3214 | 0.498 | -0.0779 | 0.7652 |
| HS 4 | 0.0822 | 0.7035 | 1.4284 | 0.1194 | -0.2524 | 0.0713 | 1.1362 | 0.0051 | -0.2257 | 0.6237 | 0.0938 | 0.7201 |
| HS 5 | 0.1349 | 0.5768 | 1.6644 | 0.0885 | -0.0530 | 0.7307 | 1.3408 | 0.002 | 0.4019 | 0.4122 | -0.2602 | 0.4328 |
| HS 6 | -0.0027 | 0.9909 | 0.7323 | 0.5566 | -0.1480 | 0.3291 | 1.8672 | <.0001 | -0.4008 | 0.4309 | -0.3006 | 0.3389 |
| HS 7 | -0.0832 | 0.7176 | 1.2464 | 0.2446 | -0.0584 | 0.7051 | 1.0637 | 0.0161 | -2.0487 | 0.0072 | -0.0261 | 0.9366 |
| HS 8 | - | - | - | - | - | - | - | - | - | - | - | - |

GP = General Practitioner

*Part I provides the coefficients (and associated p-values) of the logistic regression and Part II provides the coefficients (and associated p-values) of the gamma regression. To calculate probabilities of consumption, the following formula should be used: exp(X)/(1+exp(X)) with X=intercept + age coefficient*mean age + male coefficient * % males + HS health state coefficient. To calculate quantity of resource consumed, the following formula should be used: exp(Y) with Y=intercept + age coefficient*mean age + male coefficient * % males + HS health state coefficient*
